# Improving Spatial Resolution by Reinterpreting Dosage for Laser-Induced Breakdown Spectroscopy Imaging: Conceptualization and Limitations

**DOI:** 10.1021/cbmi.4c00045

**Published:** 2024-07-25

**Authors:** David Ken Gibbs, Maximilian Podsednik, Patrick Tapler, Maximilian Weiss, Alexander Karl Opitz, Michael Nelhiebel, Charles Derrick Quarles Jr, Silvia Larisegger, Andreas Limbeck

**Affiliations:** † TU Wien, Institute of Chemical Technologies and Analytics, Getreidemarkt 9/164, 1060 Vienna, Austria; ‡ KAI Kompetenzzentrum Automobil- und Industrieelektronik GmbH, Argentinierstrasse 8, 1040 Vienna, Austria; § KAI Kompetenzzentrum Automobil- und Industrieelektronik GmbH, Technologiepark Villach, Europastrasse 8, 9524 Villach, Austria; ∥ Elemental Scientific, Inc., Omaha, Nebraska 68122, United States

**Keywords:** Laser-Induced Breakdown Spectroscopy, Elemental Imaging, Image Quality, Data Processing, Applications, Laser Ablation, Deconvolution

## Abstract

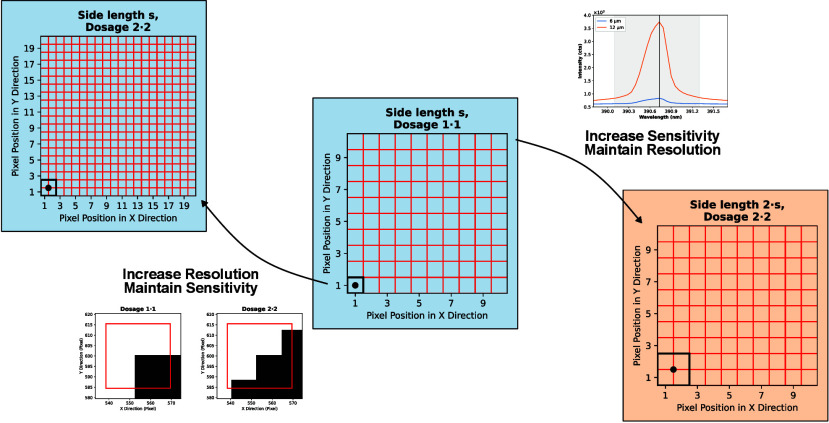

Elemental imaging in laser-induced breakdown spectroscopy is usually performed by placing laser shots adjacent to each other on the sample surface without spatial overlap. Seeing that signal intensity is directly related to the amount of ablated material, this restricts either spatial resolution (for a given excitation efficiency) or sensitivity (when reducing the laser spot size). The experimental applicability of a concept involving the spatial overlapping of shots on the sample surface is investigated and compared to the conventional approach. By systematic choice of spacing between laser shots, spatial resolution can be improved to the single digit micrometer range for a given laser spot size. Signal intensity is found to be linearly dependent on the area ablated per shot, facilitating larger signal-to-background ratios with increased spot sizes. Owing to this, the presented approach is also employed to enhance signal intensity, while preserving spatial resolution. The applicability of the method is explored by analyzing samples with distinct thickness of the surface layer, allowing for the assessment of the concept’s suitability for different sample types.

## Introduction

Due to its simple instrumentation, minimal sample pretreatment, possibility for ambient atmosphere operation, as well as multielement and quantitative capabilities, laser-induced breakdown spectroscopy (LIBS) has gained attention as a complementary technique for methods such as laser ablation-inductively coupled plasma-mass spectrometry (LA-ICP-MS), secondary ion mass spectrometry (SIMS), electron probe microanalysis (EPMA), and micro-X-ray fluorescence (μ-XRF). Especially in the context of spatially resolved analysis, LIBS can offer key advantages, e.g., light element detection and acquisition speeds of kilohertz (kHz) per pixel while achieving parts-per-million-scale sensitivity with a spatial resolution of less than 10 μm.^1−3^ As a result, LIBS has already been used for the analysis of a broad range of samples. For instance, in their review on the use of LIBS in bioimaging, Modlitbová, Pořízka, and Kaiser mention that as early as 2006 the spatial distribution of Pb and Cd in leaves was investigated with LIBS.^4^ The study of biological tissues is also possible; for instance, Sancey et al.^5^ performed multielemental mapping of Gd, Si, Fe, and Na in murine livers. In geology and archeology, LIBS is a common tool to identify, image, and quantify different minerals and elements in rocks, fossils, glasses, and extraterrestrial materials.^6^ For example, Moncayo et al. showed how the mineral phases pyrite, turquoise, and silica can be identified and differentiated based on principal component analysis.^7^ Furthermore, LIBS was employed in the semiconductor industry to assess different metallic and nonmetallic impurities as well as dopants.^8^ In material science, LIBS can also be utilized to examine elemental distributions; especially for catalytic particles this can provide valuable insights into the catalytic activity, which was demonstrated by Trichard et al.^9^

Signal generation in LIBS relies on the formation of a plasma on the sample by irradiation with a laser. A highly energetic laser pulse causes excitation of the sample and creates a highly ionized plasma consisting of cations and electrons, which emits discrete spectral lines as well as bands and continuum as a result of recombination and deexcitation processes.^10−12^ The emission is collected and analyzed by a combination of spectrometer and detector such that an emission spectrum is generated, which is unique for the sample and can then be evaluated.^13^ Spectral lines and bands are characteristic for the elements contained within the sample, since the deexcitation is a transition between discrete energy levels, thereby facilitating the identification of the sample’s constituents.^14^

Imaging is a common application of LIBS, owing to its simultaneous multielement capabilities,^11^ and some possible applications were summarized by Gardette et al.^15^ in an excellent review. Conventionally, LIBS imaging is performed in a way where each laser shot ablates a new position on the sample. As a result, for a laser system with a given energy output, the signal will depend on the amount of ablated material if the excitation efficiency is identical.^16^ If the ablation depth is then considered to be constant, the diameter of the laser spot will dictate not only spatial resolution but also emission intensity. This means that high emission intensity and spatial resolution are competing interests that must be considered for every sample.^15^ Spot sizes in the single digit micrometer range can be achieved, but then detection of the emitted photons, i.e., sensitivity, becomes an issue.^17^

In order to boost sensitivity for a given instrumentation, the LA-ICP-MS community has adopted an approach demonstrated by the group of Šala.^18,19^ They define the general case of single pulse analysis, i.e., one laser shot leads to one pixel in the image, with the parameter dosage *D* = 1. Alternatively, the laser can be scanned across the sample continuously with dosage *D* > 1. Here, *D* relates to the number of shots that are combined in the laser scanning direction in order to represent a pixel. Since multiple shots are fired on the sample to generate one pixel, the signal intensity is considerably higher. Šala et al. further explain that even though the image quality may be reduced due to blur, less noise is generated and faster imaging speeds can be realized.^18^

In principle, the concept of overlapping shots should result in systematic improvements in the quality of LIBS images too but has not yet been reported in literature. A reason for this could be the recent introduction of nanosecond pulse width excimer lasers to LIBS, which brings about major benefits with regards to LIBS imaging. For instance, even though flat-top beam profiles have also been achieved for the laser type most commonly used in LIBS, the Nd:YAG laser, they are featured more often in commercial *excimer* lasers systems.^19−22^ This allows for more representative sampling of the sample surface compared to Gaussian beam profiles. Furthermore, the Nd:YAG lasers used in LIBS are generally limited to lower repetition rates compared to excimer or femtosecond pulse width lasers, making the imaging of larger sample areas with high spatial resolution a time-consuming process.^17^

In this work, we therefore propose a redefined concept of dosage for LIBS imaging, which can improve image quality in two ways: enhancing spatial resolution while maintaining sensitivity and/or boosting sensitivity while retaining spatial resolution. First, the respective motivation for overlapping shots will be discussed, before demonstrating the applicability of the concept on different samples—comblike copper structures and ceramic thin films—to investigate requirements for the use of the concept.

## Experimental Section

### Conceptualization of Dosage in LIBS

**Figure 1:**
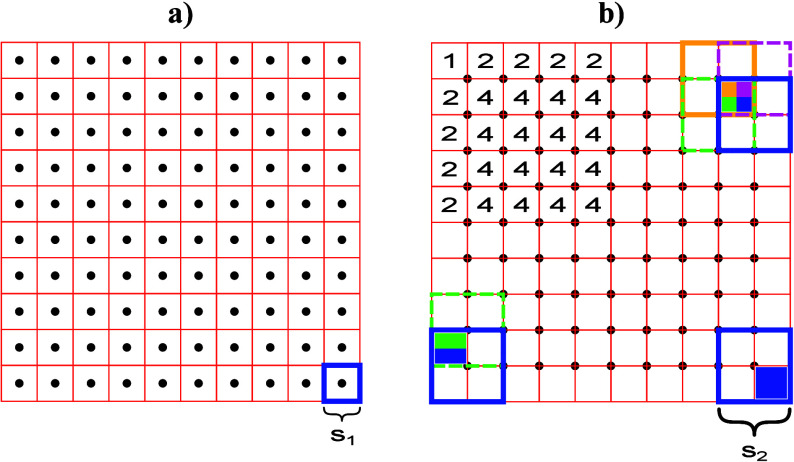
Ablation grid explaining the concept of dosage for LIBS. (a) Ablation grid for nonoverlapping shots (dosage *D* = 1•1). (b) Ablation grid for overlapping shots with dosage *D* = 2•2, also showing the three deconvolution cases (corner, rim, and center pixels) and how many shots contribute to each pixel. See text for a detailed description.

In principle, when employing dosages *D*_*x*_, *D*_*y*_ > 1, square laser shots with a spot size *s*_*x,y*_ = *s*_*x*_•*s*_*y*_ are overlapped such that their centers are separated by *s*_*x*_/*D*_*x*_ and *s*_*y*_/*D*_*y*_ in the *x*- and *y*-direction, respectively, on the sample surface. Though this concept could be applied to the *x*- and *y*-axis independently with any value of *D*_*x*_ and *D*_*y*_, this work only deals with the case where  and the overlapping of shots is performed in both directions equally, i.e., *D*_*x*_ = *D*_*y*_. In the following, the same dosage in both spatial coordinates is therefore simply denoted as *D* = *D*_*x*_•*D*_*y*_. Furthermore, square laser spots are employed to create the ablation grids/patterns. Due to the fact that the emission intensity is related to the sample quantity ablated by the laser, an increase in spot size will induce higher sensitivity (for constant ablation depth and excitation efficiency). When employing a spot size that fulfills *s*_*x,y*_ = *D*_*x,y*_·*s*_*x,y*_^*D* = 1•1^, where *s*_*x,y*_^*D* = 1•1^ is the spot size for nonoverlapping imaging (*D* = 1•1), the same amount of pixels is obtained in the final image, i.e., the spatial resolution of *s*_*x,y*_^*D* = 1•1^ is retained. This is illustrated in Figure 1, where the ablation and corresponding pixels grids are compared for (a) *D* = 1•1 and (b) *D* = 2•2 for the case that *s*_2_ = 2*s*_1_ = 2*s*_*x,y*_^*D* = 1•1^. The ablation grid is represented by the black dots, which symbolize the centers of the laser shots, while the red squares correspond to the pixel grid in the final image.

Importantly, the emission spectra of laser shots that ablate the same area of the sample must be deconvoluted to assign an intensity value to a pixel in the image. Iolite 4 (Version 4.8.9), which was used for the creation of the images in this work, performs this process automatically. In simple terms, the deconvolution step can be explained by extending the order of the position matrix by (*D*_*x*_ – 1) × (*D*_*y*_ – 1), dividing a spot into *D*_*x*_ × *D*_*y*_ pixels, and storing the signal intensity at the positions of the subdivided pixels. Repeating this process for all laser shots and calculating the average of the intensities stored at each position will then return the value that is assigned to the pixels. By comparing the pixel grids in Figure 1, it is clear that the same spatial resolution can be obtained with overlapping shots (*D* > 1•1), when the contributions of shots are deconvoluted to their respective pixels.

Figure 1b additionally demonstrates the three deconvolution cases that occur when an increased dosage is applied. In the corner case (Figure 1b, bottom right), only one shot partially covers the area that is taken up by a pixel in the final image. For the rim case (Figure 1b, bottom left), two shots overlap in the area that is represented by the pixel. The intensities of these two shots are averaged and stored in the highlighted pixel. Depending on the dosage, the rim will extend further into the image. The most frequent case in the image, the center case (Figure 1b, top right), is the result of *D* = 2•2 = 4 shots in total, which overlap within the area of the pixel. The annotated numbers in the top left quadrant of the image of Figure 1b show how many shots contribute to the pixel’s intensity value when applying dosage *D* = 2•2. By employing the proposed notation, the factors *D*_*x*_ and *D*_*y*_ resemble the amount of shots that overlap in the corresponding direction within the spot size of one laser shot, and the product *D* = *D*_*x*_•*D*_*y*_ is synonymous with the number of shots that contribute to a specific pixel in the image (except for positions at the corner and the rim).

The imaging concept proposed here is similar to the oversampling approach by Van Malderen, Van Elteren, and Vanhaecke^23,24^ for 2D image generation via LA-ICP-MS. In comparison, only flat-top beam profiles were investigated, and the deconvolution process is simpler, due to the restriction imposed on the ablation parameters (more precisely, the use of integer values for *D*_*x*_ and *D*_*y*_).

### Sample Types/Preparation

**Figure 2:**
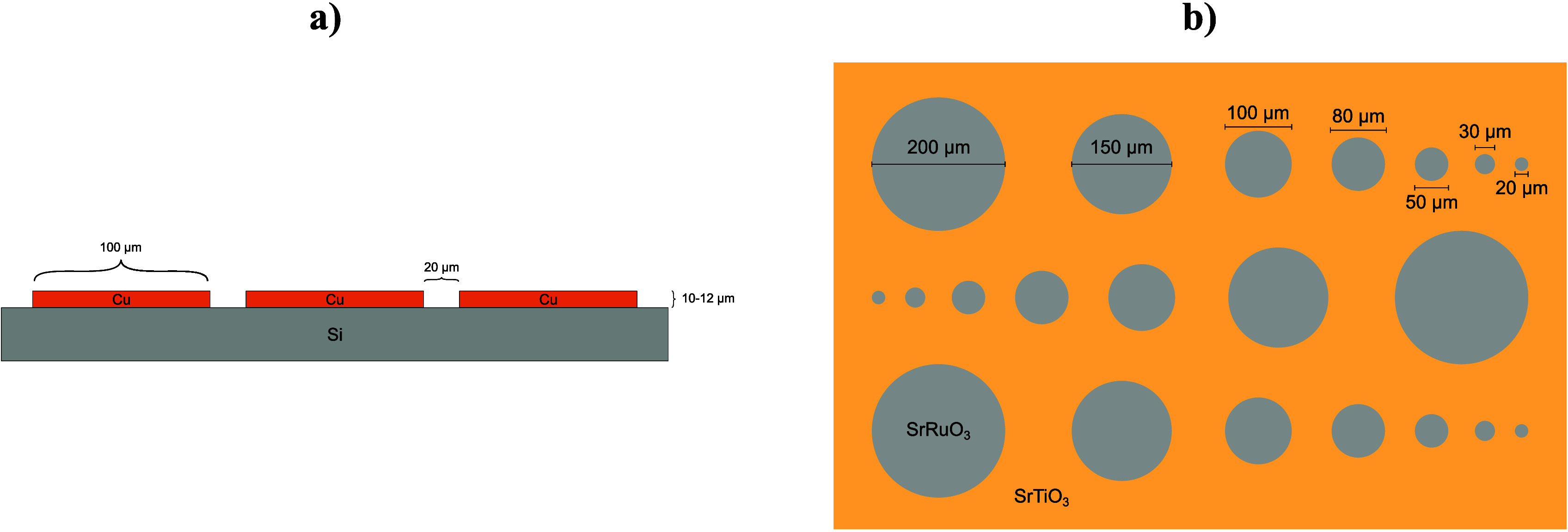
Schematic structure of the investigated samples. (a) Copper comb structures on silicon substrate. (b) Circular SrRuO_3_ patterns on SrTiO3; the thickness of SrRuO_3_ is approximately 150 nm.

For method development, the conceptualization of the dosage concept, and the creation of the images shown in this work, two sample types were investigated to show the effect of dosage on LIBS images. Copper comb structures were provided by Infineon Austria GmbH and basically consist of a silicon substrate (10 mm × 10 mm) covered by a copper layer with 10–12 μm thickness as evaluated by profilometer measurement with a “DektakXT” (Bruker, MA, USA). The copper combs themselves were approx. 100 μm wide and spaced 20 μm apart (see Figure 2a). For image generation, special attention was given to a constant sample area of approx. 1200 μm × 1200 μm and that the ablated area contained notable features, e.g., edges of combs.

For the second sample type shown in Figure 2b, SrRuO_3_ layers with a thickness of ∼150 nm (determined with the DektakXT) were deposited with a custom-built sputter device (Huber Scientific, Austria) on SrTiO_3_(100) single crystals (10 × 10 × 0.5 mm^3^, MaTecK, Germany). The sputter target was acquired from AEM Deposition, China. The SrRuO_3_ layer was structured by photolithography and subsequent argon ion beam etching of a polymer-based mask, producing circular structures with a diameter of approximately 200, 150, 100, 80, 50, 30, and 20 μm. Further information on the preparation of this sample type can be found in refs (25) and (26). The analyzed area was approximately 1200 μm × 600 μm for all images.

### LIBS Instrumentation

LIBS measurements were performed by coupling an “imageGEO193” laser ablation system from Elemental Scientific Lasers (Bozeman, MT) with a “SpectraPro HRS-750-MS” spectrometer connected to a “PI-MAX4:1024x256” Intensified Charge-Coupled Device (ICCD) camera, both from Teledyne Princeton Instruments (Acton, MA). The intensifier of the ICCD was of the Gen III *Filmless* (HBf) type. The laser was equipped with a TwoVol3 cell and an ablation cup dedicated to LIBS measurements, which allowed for two spectrometers to be coupled via optical fibers. During the measurements, one opening was closed with a plug and the other connected to the spectrometer with a fiber of 1.1 m length. The general acquisition parameters for both sample types are given in Table 1. Grating and spectral window were chosen in such a way that the emission lines of interest could be measured simultaneously with sufficient sensitivity and without saturating the ICCD detector.

**Table 1:** Acquisition Parameters for LIBS Measurements

	sample type	
parameter category	copper combs	SrRuO_3_ on SrTiO_3_
laser		
energy	10 J·cm^–1^	5 J·cm^–1^
frequency	100 Hz	100 Hz
ablation atmosphere	800 mL·min^–1^ He	800 mL·min^–1^ He
spectrometer/ICCD		
grating	600 grooves·mm^–1^	600 grooves·mm^–1^
spectral window	370.6–409.2 nm	340.6–379.2 nm
gate delay	0.1 μs	0.1 μs
gate width	10 μs	10 μs
entrance slit width	200 μm	200 μm
intensifier gain	20	20
emission wavelengths	Cu: 406.28 nm	Sr: 346.47 nm
	Si: 390.73 nm	Ru: 372.83 nm
		Ti: 376.06 nm

## Results and Discussion

Copper comb structures on silicon substrate as well as circular SrRuO_3_ patterns on SrTiO_3_ were investigated to show the applicability of overlapping shots to generate LIBS images. The former served as an example for samples that are sufficiently homogeneous regarding the depth of the sample. This allowed for the dosage concept to be employed to improve either spatial resolution or sensitivity, which is demonstrated for a specific spot diameter and a specific spatial resolution, respectively. Conversely, the SrRuO_3_/SrTiO_3_ structures exemplify the need for sample homogeneity due to the low thickness of SrRuO_3_ on the substrate.

**Figure 3:**
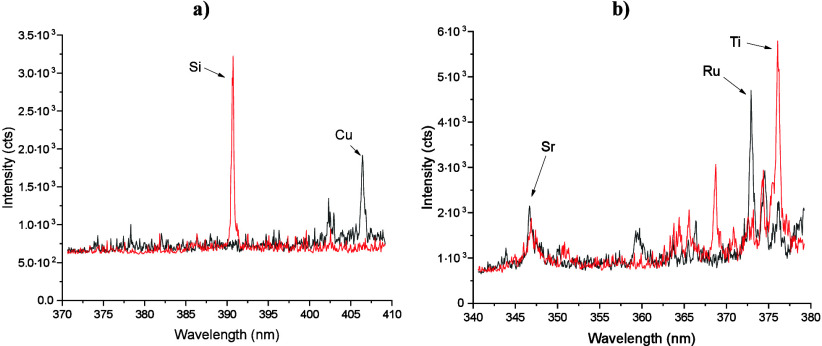
Single shot spectra on different sample areas. (a) Cu- (black) and Si-rich area (red) on the copper comb structures (spot size: 12 μm, *D* = 1•1). (b) SrTiO_3_- (black) and SrRuO_3_-rich area (red) on the circular patterns (spot size: 20 μm, *D* = 1•1). The annotated emission lines were used for image generation.

Typical single shot emission spectra (*D* = 1•1) for both the copper comb structures and SrTiO_3_/SrRuO_3_ patterns are depicted in Figure 3. The spectra show good signal-to-background-ratios (SBRs) for all observed emission lines. As mentioned before, the spectral windows were chosen to obtain sufficient intensity for the elements of interest without saturating the detector. With iolite 4, background-corrected signal intensities were calculated for every shot and then stored at the coordinates of the shot in separate channels for every set of boundaries that was defined by the user. By using a preset containing the parameters (baseline boundaries, integration boundaries, and center wavelength) for every element, the initial data processing step was automated.

### Using Dosage to Improve Spatial Resolution for Constant Sensitivity

Overlapping shots produced by a square/rectangular aperture in a targeted manner, where *s*_*x*_ = *D*_*x*_•*s*_*x*_^*D* = 1•1^ and *s*_*y*_ = *D*_*y*_•*s*_*y*_^*D* = 1•1^ hold and the centers of the laser shots are spaced *s*_*x*_/*D*_*x*_ and *s*_*y*_/*D*_*y*_ apart in the horizontal and vertical direction, respectively, will inevitably lead to a rise in the amount of pixels in the final image, if an appropriate deconvolution step is performed. Compared to nonoverlapping imaging (*n*^*D*^^=1•1^), the number of pixels *n* in the image generated by applying a dosage *D* = *D*_*x*_•*D*_*y*_ can be calculated according to *n* = *D*_*x*_•*D*_*y*_•*n*^*D*^^=1•1^. An increased dosage results not only in a larger number of pixels, thereby improving counting statistics, but also in a smoother representation of borders between regions of high and low intensity as a consequence of the smaller pixel size.

**Figure 4:**
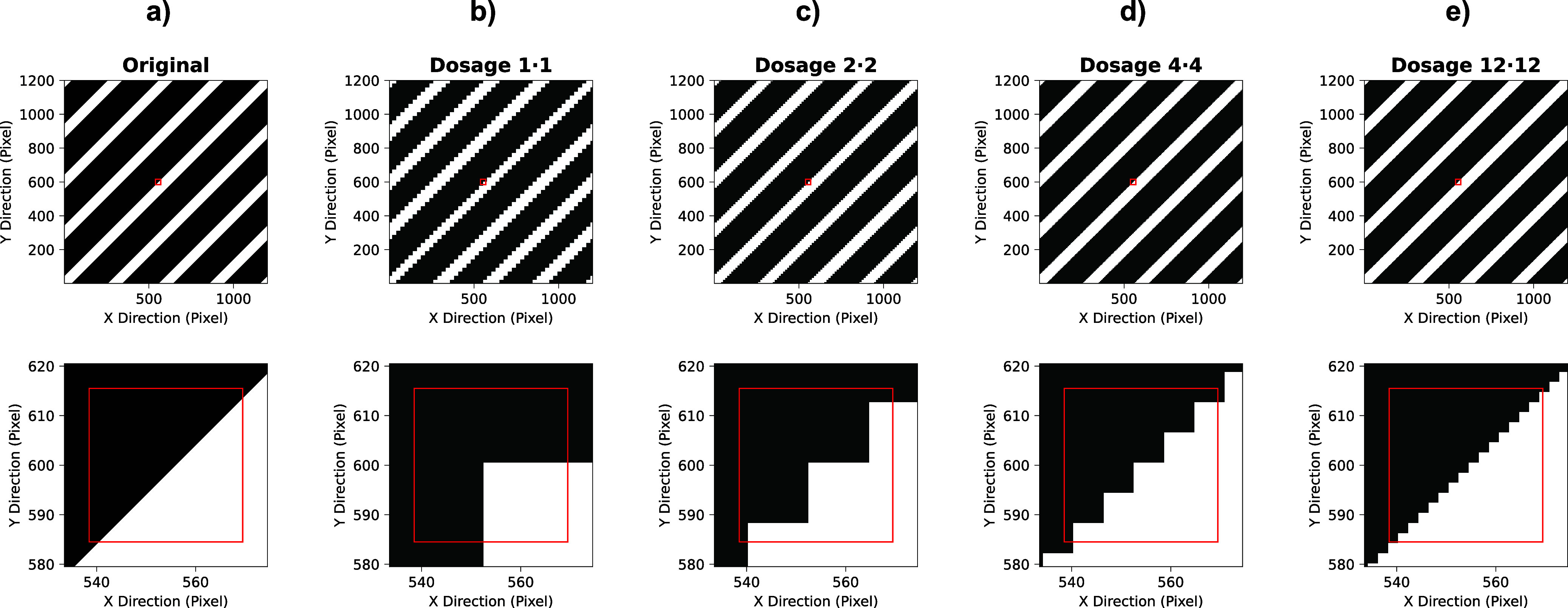
Simulated heatmaps visualizing the sampling of a periodic structure with different spatial resolutions. The top row shows an overview of the heatmaps and the bottom row a magnification of a border region. (a) Original periodic structure resembling the investigated copper comb structures. (b–e) Resulting heatmap when applying (b) *D* = 1•1, (c) *D* = 2•2, (d) *D* = 4•4, and (e) *D* = 12•12.

This principle is demonstrated in Figure 4, which shows simulated heatmaps of a periodic structure that is similar to the copper combs investigated in this work. A sample is assumed to contain two elements: one element that appears as black and the other as white. The *original*, which represents the sample surface before ablation, features a smooth transition from the black to the white regions. For simplicity, the ablated sample area is assumed to the have dimensions of 1200 × 1200 pixels. If the sample is then ablated with a square aperture with a spot size of 24 × 24 pixels and dosage *D* = 1•1, the borders in the corresponding intensity heatmap (Figure 4b) will appear jagged, considering that there is an insufficient amount of pixels that describe the border. This is shown even clearer by the bottom row of Figure 4b, where compared to approximately half of the magnified area in the original (Figure 4a), only one-fourth is represented as white. By keeping the spot size at 24 × 24 pixels but increasing the dosage to *D* = 2•2 (Figure 4c), *D* = 4•4 (Figure 4d), or even *D* = 12•12 (Figure 4e), this mismatch of black and white area is significantly reduced, allowing for a better representation of the sample in the generated LIBS image. Conceptually, the boost in the number of pixels can be achieved by using smaller spot sizes too but would be accompanied by a loss of sensitivity, which is crucially counteracted when employing the dosage concept.

**Figure 5:**
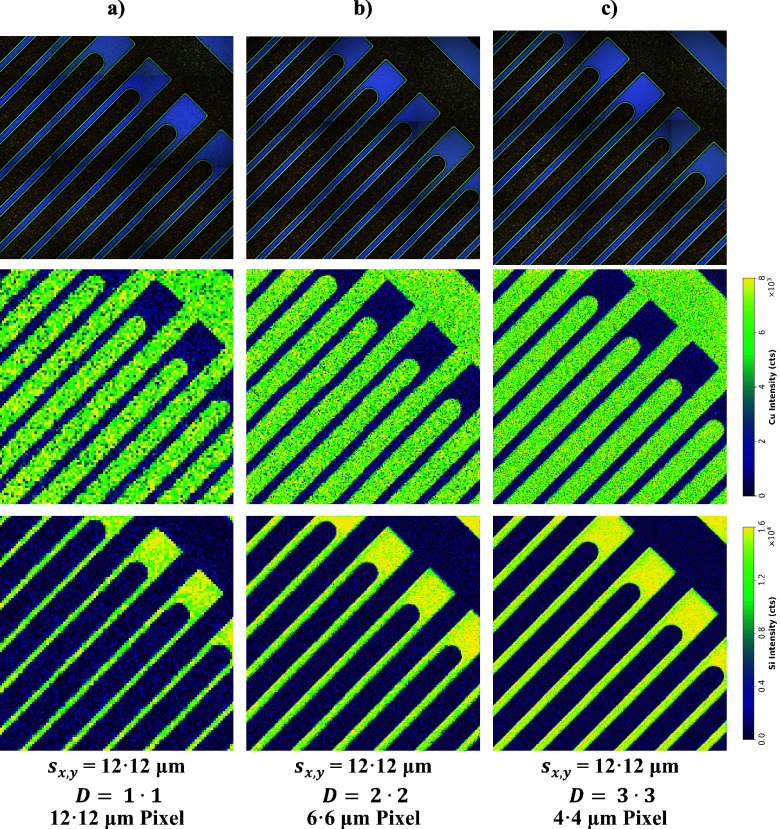
Utilizing dosage to improve spatial resolution. Cu and Si images were acquired with a constant laser spot size of 12 μm and variable dosage: (a) *D* = 1•1, (b) *D* = 2•2, and (c) *D* = 3•3.

To show the applicability and effectiveness of the dosage concept for enhancing spatial resolution, Cu and Si images of the copper comb structure were acquired, which are illustrated in Figure 5. For this, a spot size of 12 μm with different dosages (*D* = 1•1, 2•2, and 3•3) was used, resulting in a pixel size of 12, 6, and 4 μm, respectively. The sampled area was approximately 1200 μm × 1200 μm in size—resulting in images with 100 × 100, 200 × 200, and 300 × 300 pixels—and was ablated in 6, 13, and 25 min. While the images with *D* = 1•1 appear (subjectively) pixelated, an increase of dosage results in (subjectively) smoother borders. This is particularly notable when comparing the Cu images with *D* = 1•1 and *D* = 3•3, where the latter exhibits significantly better agreement with the visual appearance of the sample. Furthermore, it can be seen that, regardless of dosage, the sensitivity is approximately equal for all images.

**Figure 6:**
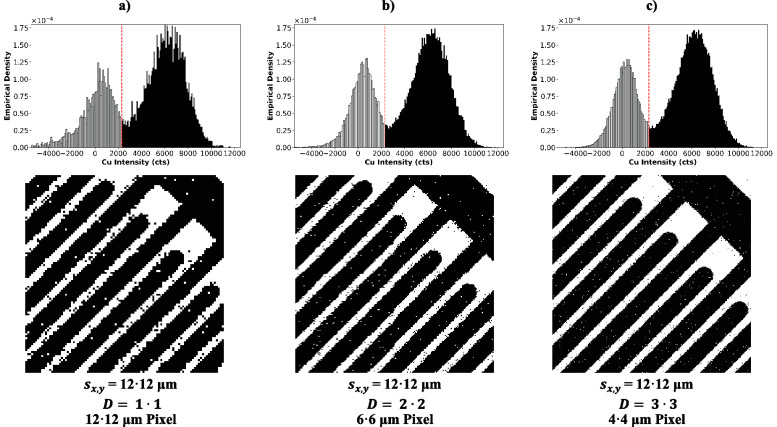
Image comparison based on binary Cu intensity. The middle row shows the empirical density histogram of the Cu intensity of the heatmaps in Figure 5 with the threshold used for binarization (red); the bottom row displays the binarized Cu intensity heatmaps. The images were acquired with a constant laser spot size of 12 μm while applying (a) *D* = 1•1, (b) *D* = 2•2, and (c) *D* = 3•3.

Though the improvements are visible in the color images, the image quality comparison can be facilitated by conversion into binary images based on the evaluation of intensity histograms.^21,27,28^ In Figure 6 the histograms and binary images of Cu of Figure 5 are depicted. The vertical axis shows the empirical density, which corresponds to the frequency of the Cu intensity values within the intensity range (bin) divided by the total number of intensity values (i.e., the amount of pixels in the image) and the bin width. This ensures that the integral of the histogram is equal to 1 for all histograms and that different bin widths are compensated. Hence, direct comparison of the histograms regardless of the number of pixels in the image is possible. All three histograms exhibit two Gaussian distributions for regions containing some or no copper, which overlap slightly, and appear smoother with increased dosage, due to the improved counting statistics. In an attempt to assign the copper-containing class to all pixels that contain an intensity belonging to the Gaussian distribution at higher intensity, the threshold for image binarization was set arbitrarily to the same value for all images. Setting aside the exact choice of the threshold, the binary images in the bottom row of Figure 5 are clearly less pixelated and feature significantly smoother transitions from black to white when employing higher dosages.

### Employing Dosage to Enhance Sensitivity for Constant Spatial Resolution

**Figure 7:**
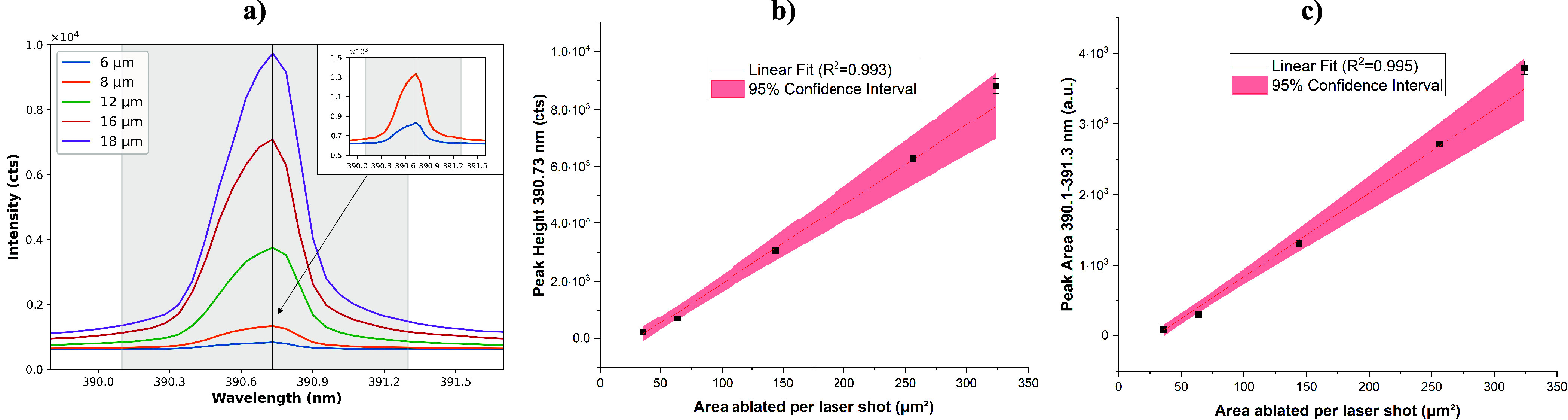
(a) Comparison of the Si emission for different laser spot sizes (averaged from 100 shots); the vertical, black line marks the intensity maximum; the shaded area shows the boundaries for the intensity integral. (b) Linear regression of the peak height (intensity maximum at 390.73 nm) against the ablation area based on five sets of 20 shots. (c) Linear regression of the peak area (integral from 390.1 to 391.3 nm) of the silicon emission against the ablation area based on five sets of 20 shots.

Since emission intensity is directly proportional to the number of excited atoms in the analytical volume, for a given ablation depth the LIBS signal intensity will highly depend on the chosen laser spot size. Hence, sensitivity will improve when choosing a larger spot size; simultaneously, spatial resolution will suffer, if imaging experiments are performed in the conventional, nonoverlapping manner. The dependency of the Si emission intensity on the laser spot size is illustrated in Figure 7a for 6–18 μm. In this region, the Si intensity was in fact experimentally found to be linearly dependent on the spot size, and an increase in SBR with spot size can be observed. Evaluating both the peak height at 390.73 nm (Figure 7b) as well as the peak area at 390.1–391.3 nm (Figure 7c) and plotting against the area ablated by every shot yielded excellent linear fits (*R*^2^ > 0.99).

**Figure 8:**
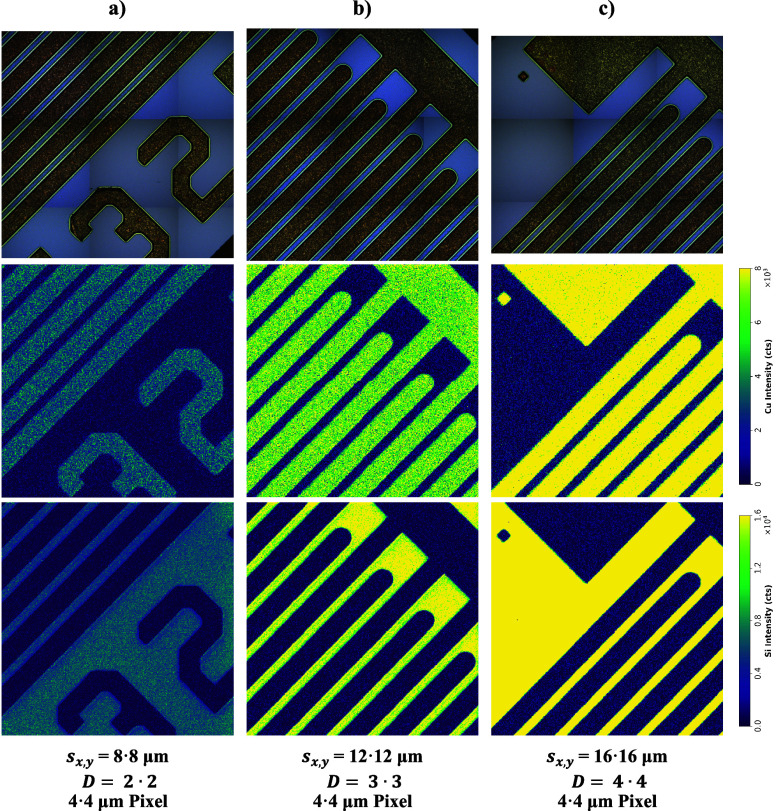
Employing dosage to improve sensitivity. Cu and Si images were acquired with coordinated laser spot size and dosage to generate a pixel size of 4 μm: (a) 8 μm spot size and *D* = 2•2, (b) 12 μm spot size and *D* = 3•3, and (c) 16 μm spot size and *D* = 4•4.

Based on this result, dosage was utilized to systematically boost sensitivity for constant spatial resolution as depicted in Figure 8. Spot sizes of 8, 12, and 16 μm with *D* = 2•2, 3•3, and 4•4, respectively, were employed to generate images with a spatial resolution of 4 μm. An area of approximately 1200 μm × 1200 μm was sampled, thereby generating images with 300 × 300 pixels, which were ablated in 25 min. Since the intensity scales are equal for all images, it is clear that emission intensity increases with spot size.

**Figure 9:**
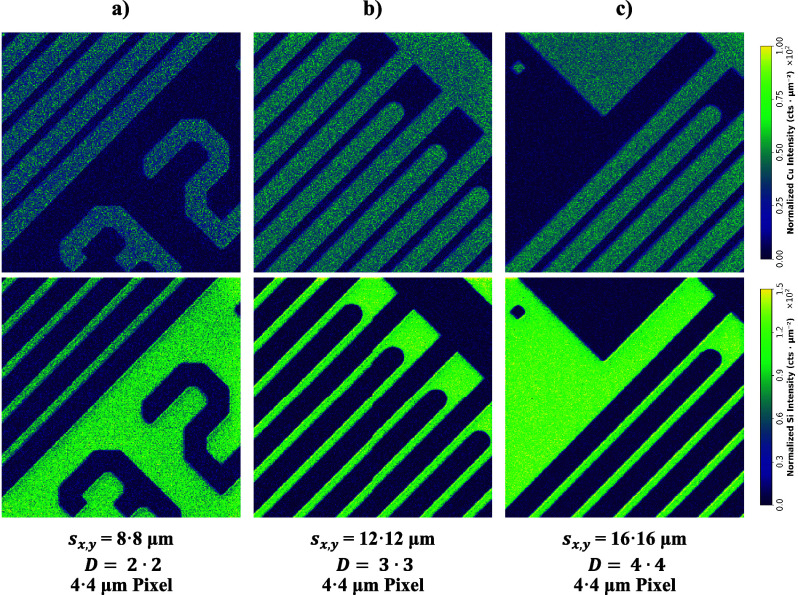
Applying area-normalization to make images with distinct sensitivities comparable. The intensities of the images in Figure 8 were normalized to the spot area to show that the image quality is approximately equal regardless of the spot size. Employing (a) 8 μm spot size and *D* = 2•2, (b) 12 μm spot size and *D* = 3•3, and (c) 16 μm spot size and *D* = 4•4 resulted in a pixel size of 4 μm.

To better compare image quality with distinct sensitivities, the intensity was then normalized to the area of the laser spot used, which can reasonably be performed following the previous discussion of Figure 7. The area-normalized images of Figure 8 are illustrated in Figure 9. While the image quality/sharpness of borders appear (subjectively) equal for all spot sizes, the normalized signal intensity for the images with 8 μm spot size is slightly reduced. This can be explained by the aggravated relative error of area normalization for lower spot sizes or alternatively by the low SBR for 8 μm compared to, for instance, 16 μm (cf. Figure 7a).

**Figure 10:**
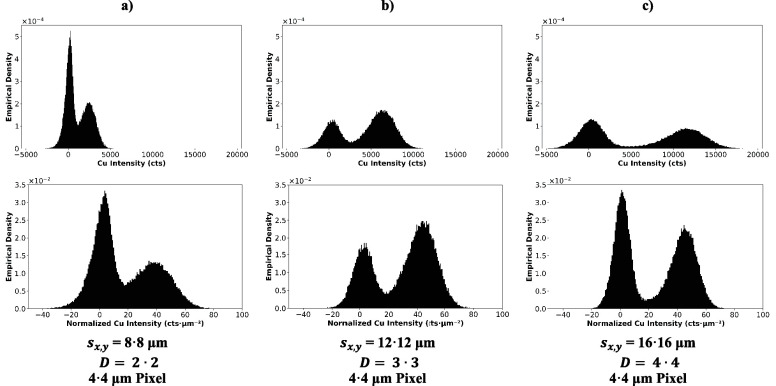
Empirical density histograms based on regular Cu intensity (top) and area-normalized Cu intensity (bottom) for a constant pixel size of 4 μm. The underlying heatmaps (cf. Figure 8 and Figure 9) were acquired by using (a) 8 μm spot size and *D* = 2•2, (b) 12 μm spot size and *D* = 3•3, and (c) 16 μm spot size and *D* = 4•4.

Similar to the previous chapter, the Cu intensity histograms can provide valuable insight into how the images change with dosage. Figure 10 illustrates the empirical density histograms of the Cu intensity images in Figure 8 (non-normalized, top row) and Figure 9 (area-normalized, bottom row). Naturally, the maxima of the Gaussian distribution containing some (with the mean of the distribution at high intensity) and no copper (with the mean at low intensity) will depend on the investigated area on the sample. Nevertheless, the histograms of the non-normalized data show a clear trend to an improved separation of the Gaussian distribution. This can again be explained by the increased sensitivity with larger spot sizes. The histograms of the area-normalized intensity feature distributions with a similar mean and width, further substantiating that area-normalization can be applied with high certainty in this context to compare images with different sensitivities.

### Exploring the Limitations of Dosage in LIBS

Until now, multiple shots could be placed on the same area on the copper combs without exceeding the surface layer, and the dosage concept could be employed without restrictions. This would also apply to any sample, e.g., geological material, that has a layer thickness in the micrometer range. Issues arise when the layer of interest is thinner than the total ablation depth, which in theory can be calculated from the product of the (assumed to be constant) ablation depth per shot and the number of shots at each pixel location, which in turn is equal to the dosage *D* = *D*_*x*_•*D*_*y*_.

The laser parameters used for the copper comb structures (cf. Table 1) resulted in an ablation rate per shot in the region of 200 nm. In that case, even for the maximum dosage *D* = 2•2 (i.e., 16 shots for one pixel), the total ablation depth was below the layer thickness of Cu on the Si substrate (10–12 μm). However, the thickness of SrRuO_3_ on the SrRuO_3_/SrTiO_3_ structures, which were examined as a second application example, is significantly lower (∼150 nm). To show the limitations of LIBS dosage for such samples, an initial image with *s* = 10 μm and *D* = 1•1 was created. Compared to the copper combs, the ablation depth had to be reduced by lowering the laser energy (in order to not ablate deeper than the SrRuO_3_) and amounted to approximately 120 nm. It is important to mention here that the use of the excimer laser instead of a nanosecond pulse width Nd:YAG LIBS laser is crucial to analyze these thin film structures. Considering that the ablation depth per shot can be in the micrometer range for Nd:YAG systems, it would not have been possible to ablate only the SrRuO_3_ without significant intensity from the Ti in the substrate, even in nonoverlapping imaging.

**Figure 11:**
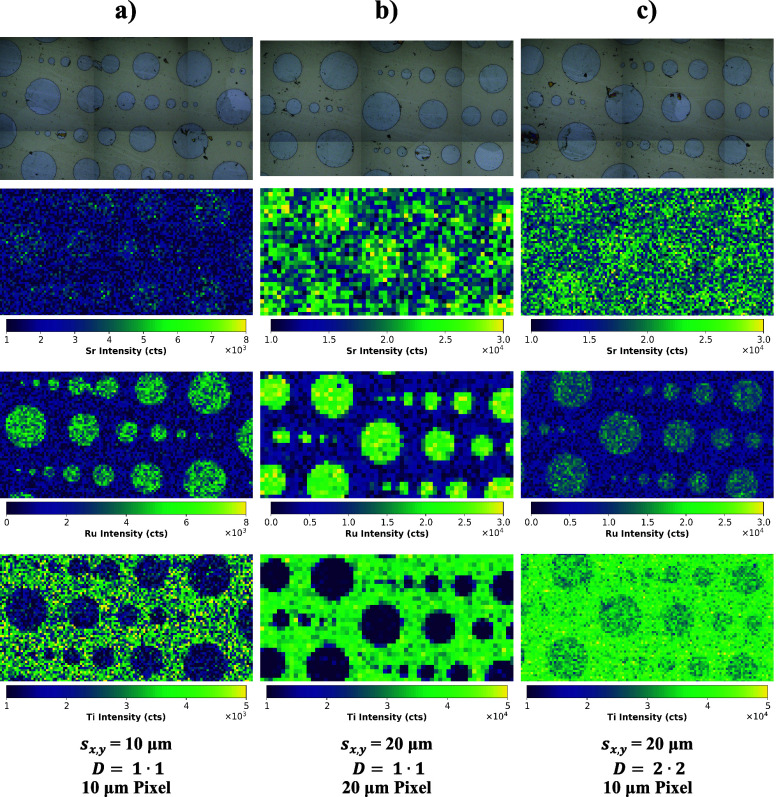
Images of the SrRuO_3_/SrTiO_3_ structures obtained with different laser parameters: (a) 10 μm spot size and *D* = 1•1, (b) 20 μm spot size and *D* = 2•2, and (c) 20 μm spot size and *D* = 2•2. The heatmaps illustrate the conflict between spatial resolution and sensitivity, particularly for samples with low depth homogeneity.

It can be seen that the images in Figure 11a contain comparably large amounts of noise, with the most extreme case being Ti, because there the SBR is insufficient. Hence, a larger spot size would be desirable, as depicted in Figure 11b for *s* = 20 μm and *D* = 1•1, but then the spatial resolution is unsatisfactory and the smallest SrRuO_3_ circle (with a diameter of 20 μm) is only represented by a singular pixel. Ideally, the spatial resolution of 10 μm spot size (or smaller) and the sensitivity of 20 μm would be combined by utilizing the dosage concept. This was attempted for Figure 11c by using *s* = 20 μm and *D* = 2•2, and indeed the improvement in resolution can be achieved. However, the Ti image in Figure 11c reveals that the sample surface is not represented correctly. Compared to nonoverlapping imaging, significant Ti intensity is present in areas which should only contain SrRuO_3_ The reason for this is the intensity averaging/deconvolution, since SrTiO_3_ will make up a notable portion of the total ablated material, when more than one shot is fired at the same area on the surface. Apart from Ti this effect can be observed for Ru too, where compared to *D* = 1•1 the Ru intensity is markedly decreased.

In short, as soon as dosage *D* > 1 is applied and multiple shots are (partially) fired at the same area, the depth/thickness of the investigated layer becomes limiting and mixing/averaging of different layers occurs. This is not surprising, considering that it was discussed by Šala for overlapping shots^18^ and by Van Malderen, Van Elteren, and Vanhaecke for their 2D image deconvolution approach^23,24^ in LA-ICP-MS. Hence, it is important to assess whether depth resolution is required or a surface-near layer can be ablated, where the thickness of the ablated layer will depend on the total ablation depth. Since the ablation depth depends on the laser parameters used, lower layer thicknesses could be analyzed by reducing the laser energy, for example, if sensitivity is sufficient.^23^

## Conclusions

In this work a concept for LIBS imaging was demonstrated, with which either the spatial resolution (for a given spot size) or the sensitivity (for a given spatial resolution) can be improved. By means of a 193 nm excimer laser combined with a high-resolution spectrometer and ICCD detector, images were created of different sample types, which differ in structure and layer thickness, to demonstrate how image quality may be enhanced for a given sample by applying a systematic spatial overlap of laser shots on the sample surface. The images generated for the copper comb structures exemplify the applicability of the approach to different elements, seeing that both Cu and Si show significant improvements with increased dosages. As indicated previously, the main drawback of the demonstrated LIBS dosage concept is the need for depth homogeneity. If *D*_*x*_ and *D*_*y*_ are larger than 1 and are integer numbers, a minimum of *D* = 2•2 = 4 shots are fired at the same area of the sample, resulting in a significant contribution of nonsurface material to the signal intensity of the surface representation. This can be adequate for geological or even biological thin sections, where the composition does not change or varies negligibly in terms of the depth, but inappropriate for thin layers produced by physical vapor, chemical vapor, or pulsed laser deposition. Therefore, layer thickness/depth homogeneity need to be considered ahead of applying the dosage concept for imaging. Depending on the desired information, however, it may be more adequate to perform depth-profiling analysis, where signal intensity is recorded as a function of the ablated depth, instead of imaging.

It is important to reiterate that the excimer laser system used for the application of the demonstrated approach is beneficial, since the high repetition rate and square aperture enabled the application of the dosage concept in the first place. Additionally, even though solid-state lasers with high pulse energies lead to an improved sensitivity compared to the excimer laser, the ablation rate can be in the micrometer range for 1064 nm Nd:YAG lasers,^29^ restricting the layer thickness that can be analyzed using the dosage concept.

Even though this was not the intention of this work, the proposed dosage concept in theory results in a reduced acquisition time. As a comparison, consider an image acquired with no overlapping and consisting of 100 × 100 pixels, equivalent to 100 × 100 laser shots. To achieve the same image dimensions with applied dosage, fewer laser shots are required. In general, (*n*_*x*_ – *D*_*x*_ + 1) × (*n*_*y*_ – *D*_*y*_ + 1)) shots are necessary, where *n*_*x*_, *n*_*y*_ are the number of shots in the *x*- and *y*-direction for the nonoverlapping image, to generate an image with the same number of pixels, assuming that *D*_*x*_ and *D*_*y*_ are integer values. Applying *D* = *D*_*x*_•*D*_*y*_ = 2•2, this means that only 9 801 shots instead of 10 000 are required for the same sample area. For high repetition rates (e.g., 100 Hz) the time saved is less than 2 s, but for lower repetition rates this could reduce analysis time significantly.

Finally, it is essential to mention that in this work dosage was utilized for main constituents of the sample, which also deliver high signal intensity. When analyzing traces, sensitivity may become limiting if the emission intensity is low in the first place, i.e., for elements with low excitation efficiency. However, the challenge then is mainly detection of the emitted photons, which can be addressed with changes in spectrometer setup or—like for the setup used in this work (high-resolution spectrometer and ICCD)—by adjusting the spectral window that is measured.
